# Lysyl Oxidase Promotes the Formation of Vasculogenic Mimicry in Gastric Cancer through PDGF-PDGFR Pathway

**DOI:** 10.7150/jca.92192

**Published:** 2024-02-04

**Authors:** Yifan Liu, Bojian Sun, Yuan Lin, Hong Deng, Xinyi Wang, Chuanhao Xu, Kaibo Wang, Nan Yu, Rongqing Liu, Mei Han

**Affiliations:** 1 Department of Pathogenic Biology and Immunology, School of Basic Medical Sciences, Ningxia Medical University, Ningxia, P.R. China.; 2 Department of Rheumatology and Immunology, The First Hospital of Hebei Medical University, Shijiazhuang, Hebei, P.R. China.; 3 Department of Rheumatology and Immunology, The General Hospital of Ningxia Medical University, Ningxia 750004, P.R. China.

**Keywords:** Lysyl oxidase, Vasculogenic mimicry, Gastric cancer, Platelet-derived growth factor receptor

## Abstract

**Objective:** Vasculogenic mimicry (VM) generates an important supplementary form of blood supply in cancer, which many factors regulate. However, the effect of lysyl oxidase (LOX) on VM formation is unclear. In this study, gastric cancer tissues and cells were used to investigate the role of LOX in the formation of VM.

**Materials and Methods:** The samples were collected from 49 patients with a final diagnosis of gastric cancer. According to metastasis (including lymph node metastases and distant metastases), gastric cancer samples were divided into metastasis and non-metastasis groups. Based on the degree of invasion, gastric cancer specimens were divided into T1 + T2 and T3 + T4 groups. The relative expression of LOX was detected using Western blot. The formation of VM was measured by double staining with CD34 and Periodic acid-Schiff (PAS) in gastric cancer tissue slices, and the correlation between LOX and VM was analyzed with Pearson's correlation analysis. Gastric cancer cell line BGC-803 was treated with LOX, β-aminopropionitrile (BAPN, an inhibitor of LOX), and AG1295 or AG1296 (inhibitors of the platelet-derived growth factor receptor). The formation of VM was then measured using PAS staining. The expression of platelet-derived growth factor receptor (PDGFR)α and PDGFRβ in gastric cancer cells was detected by Western blot.

**Results:** In gastric cancer samples, the level of LOX was higher in the metastasis group than in the non-metastasis group (*P* < 0.05) and in the T3 + T4 group than in the T1 + T2 group (*P* < 0.05). VM formation was greater in the T3+T4 group than in the T1+T2 group (*P* < 0.05) and in the metastasis group than in the non-metastasis group (*P* < 0.05). The expression level of LOX was positively correlated with VM formation (*P* < 0.01). In gastric cancer cells, LOX concentration was positively correlated with the degree of VM, and BAPN concentration was negatively correlated with the degree of VM (*P* <0.05). PDGFR levels in the T3+T4 and metastasis groups were relatively higher (*P* <0.01) and positively correlated with LOX levels in gastric cancer specimens (*P* < 0.01)**.** The relative expression of PDGFRα and PDGFRβ in gastric cancer cells was up-regulated with increasing LOX and downregulated with increasing BAPN (*P* < 0.05). With inhibition of PDGFRα and PDGFRβ using AG1295 or AG1296, VM formation in gastric cancer cells decreased (*P* <0.05), but the number of VM structures increased while LOX was added (*P* < 0.05).

**Conclusion:** LOX partially promotes the formation of VM in gastric cancer through the PDGF-PDGFR signaling pathway.

## 1. Introduction

Gastric cancer is one of the leading causes of cancer death worldwide, and its high mortality rate is mainly attributed to its invasiveness and metastasis 1-4.

Adequate blood supply is essential to provide nutrients for malignant tumors in the processes of invasion and metastasis. Therefore, inhibition of tumor angiogenesis to inhibit tumor growth and metastasis has become one of the main directions of antitumor therapy. Despite some positive results of antiangiogenic therapies in recent years, a deep understanding of tumor blood vessels and their role as targets for antitumor therapeutic interventions is still lacking.

Vasculogenic mimicry (VM) has been identified as a key factor in cancer progression 5. VM is the process of tumor cell-dependent angiogenesis stimulation in the body through tumor cell deformation and remodeling of the local extracellular matrix (ECM) to form blood vessels connected to the original blood vessels to ensure the nutritional supply of the tumor 6. Unlike conventional tumor angiogenesis, VM is a tumor-specific blood supply system that provides endothelium-free blood supply to tumor cells 7. VM is closely related to the biological behavior of various malignant tumors, such as growth, invasion, and metastasis 8-11. The presence of VM is a predictor of a poor tumor prognosis 12-13.

Lysyl oxidase (LOX) is present in ECM and plays an important role in converting collagen and elastin from soluble monomers to insoluble fibers. LOX is essential to maintain the structural and functional stability of ECM during the development of tissues and a functional vascular system 14-15. LOX is highly expressed in gastric cancer tissues, and its expression is significantly and positively correlated with the stage of gastric cancer, thus affecting the overall survival of patients 16-17. The ECM maintains the microenvironment for tumor cell growth through a dynamic balance of metabolic renewal and remodeling of degeneration. However, the effect of LOX on VM has not been clarified.

The platelet-derived growth factor (PDGF) signaling pathway is closely related to biological behaviors such as cell proliferation, transformation, invasion, metastasis, and angiogenesis 18-21. The platelet-derived growth factor receptor (PDGFR) is a major component of the PDGF signaling pathway. It binds directly to PDGF and has two subunits, PDGFRα and PDGFRβ. Lucero et al. 22 showed that LOX activity is a prerequisite for PDGFR-mediated chemotaxis. Inhibition of LOX activity by β-aminopropionitrile (BAPN, an inhibitor of LOX) negatively regulates PDGF-mediated chemotaxis and significantly reduces the signal transduction pathway. This discovery is an important complement to the LOX function.

To explore the effect of LOX on VM formation and its mechanism, this study collected fresh tumor samples from patients with gastric cancer. It analyzed the correlation between LOX expression and VM formation in gastric cancer tissues. Cultured gastric cancer cells were used to detect whether LOX affected VM formation and analyze the relationship between LOX and PDGFR. This study will provide new clues for LOX as a target for clinical antitumor therapy and prediction of cancer prognosis.

## 2. Materials and methods

### 2.1 Gastric cancer samples and cells

This study was approved by the Ethics Committee of Ningxia Medical University (Yinchuan, China; registration number 2018-004). All patients signed a written informed consent form before participating in this study.

#### 2.1.1 General information about the patients

The samples were taken from 49 patients with gastric cancer who underwent surgical resection of gastric tumors, including 39 men and 10 women, with an average age of 59.02 ± 11.53 years. Inclusion criteria were a diagnosis of gastric cancer before surgery, confirmed by histopathological examination after surgery, and no history of other malignant tumors. The exclusion criteria were patients who received adjuvant preoperative therapy, such as radiation therapy or chemotherapy.

Gastric cancer samples were classified according to the 2010 tumor node metastatic classification criteria of the Union for International Cancer Control (UICC). T1 refers to tumor invasion of the mucosa and submucosa, T2 refers to tumor invasion of the muscle layer or subserosa, T3 refers to tumor penetration of the serosa, and T4 refers to tumor invasion of adjacent structures or lumens into the esophagus and duodenum. The 49 clinical samples were divided into the T1+T2 group (n = 8) and the T3+T4 group (n = 41).

According to the presence or absence of local lymph node metastasis and distant metastases, gastric cancer specimens were divided into a metastatic group (40 cases) and a nonmetastatic group (9 cases).

#### 2.1.2 Cell culture

The BGC-803 gastric cancer cell line, which was derived from poorly differentiated gastric adenocarcinoma, was purchased from Shanghai Institutes for Biological Sciences, China. Cells were incubated with RPMI-1640 medium (Gibco, Carlsbad, CA) containing 10% fetal bovine serum in an incubator containing 5% CO_2_ at 37°C.

### 2.2 Immunohistochemistry

Specimens from gastric cancer patients were fixed with 10% formalin, embedded in paraffin, and sliced. The sections were then dewaxed in xylene and rehydrated in gradient alcohol. Antigen recovery was performed with citric acid buffer at 95°C for 15 min. Subsequently, the sections were exposed to 0.3% hydrogen peroxide in methanol to inactivate endogenous peroxidases. Sections were blocked with 10% normal rabbit serum for 15 min and incubated overnight with rabbit anti-LOX primary antibodies (1:100 dilution, Abcam). After incubation in horseradish peroxidase-labeled anti-rabbit IgG (HRP), sections were colored with a 3,3'-diaminobenzidine tetrachloric acid (DAB) solution and counterstained with hematoxylin solution before sealing.

The results were observed under an Olympus microscope at 400× magnification and photographed by DP controller software. At least five fields of view were selected for image analysis from each tissue section. Image J software was used for image analysis to detect the positive staining area and the accumulated optical density of the image. The relative expression of the LOX protein was measured by mean optical density (MOD). MOD = cumulative optical density/positively stained area.

### 2.3 Detection of VM in gastric cancer tissues

Gastric cancer sections were incubated with rabbit anti-CD34 antibodies (1:1,000 dilution, Abcam) overnight, followed by washing and subsequent incubation with HRP-labeled anti-rabbit IgG. The sections were stained with DAB. After periodic acid-Schiff staining (PAS, Beijing Solarbio Science & Technology Co., Ltd.), the nucleus was stained with hematoxylin and covered with cover a glass coverslip.

Sections were observed under a microscope, and images were taken. VM produced lumen-like structures formed by tumor cells with negative CD34 and positive PAS staining, with or without red blood cells in the lumen. Five field-of-views were selected in the tumor nest to avoid blood vessels, the VM structures were counted, and the average value was calculated.

### 2.4 Intervention of gastric cancer cells and detection of VM

The VM were analyzed by two-dimensional and three-dimensional culture of gastric cancer cells in vitro. For two-dimensional VM detection, cells were inoculated in 24-well plates at 10^5^ cells /well. After 24 h of culture, the culture medium was replaced with RPMI-1640 containing BAPN (Sigma Aldrich, St. Louis, MO) at different concentrations (0, 0.1, 0.2, and 0.3 mM), or containing LOX at different concentrations (0, 2.5, 5, and 10 nM) every 24 h for 3 consecutive days. For three-dimensional VM detection, the culture medium containing matrigel was added to the 24-well plate first, and then added to the cells 30min later. Incubate for 24 h, the medium containing BAPN or LOX was replaced each well every 24 h for 4 consecutive days.

PAS staining was performed according to the manufacturer's instructions. PAS staining was considered positive when the gastric cancer cells' glycogen turned purple in the ECM. The luminal structure formed by three or more connected PAS-positive gastric cancer cells could be considered a VM 11. Five high-power fields were selected, the number of VM was counted, and the average value was calculated.

The effects of PDGFRα inhibitors (AG1295) at different concentrations (0, 0.65, 1.25, 2.5, 5, 10 μM) and PDGFRβ inhibitors (AG1296) at different concentrations (0, 0.65, 1.25, 2.5, 5, 10 μM) on VM formation of gastric cancer cells were examined using the two-dimensional VM detection.

### 2.5 Western blot analysis

Freshly excised cancer specimens were taken for Western blot analysis. BGC-803 cells (10^5^ cells/well) were plated in 24-well plates and cultured for 24 h. After removing the medium, RPMI-1640 containing 0.1, 0.2, and 0.3 mM BAPN, or 2.5, 5, and 10 nM LOX was added and incubated for 24 h.

According to the manufacturer's instructions, total protein extract from the specimens and cultured cells was obtained using the total protein extraction kit (Key GEN Bio TECH, Jiangsu Province, China). After 10% polyacrylamide gel electrophoresis, isolated proteins were transferred to PVDF membranes (EMD Millipore, Billerica, MA) and further blocked in a 5% skim milk solution at room temperature for two hours. They were incubated independently with 1:100 rabbit anti-LOX, 1:200 rabbit anti-PDGFR, 1:50 rabbit anti-PDGFRα, or 1:50 goat anti-PDGFRβ antibody (Santa Cruz Biotechnology), or 1:1000 rabbit anti-actin (Key GEN Bio TECH, Jiangsu Province, China) at room temperature for 3 h and then treated with HRP-labeled anti-rabbit or anti-goat IgG. After DAB staining, the gray value of the target protein bands was quantified by Quantity One software, and the relative protein expression was calculated as follows: protein relative expression = target protein expression intensity/β-actin protein expression intensity.

### 2.6 Statistical analysis

The SPSS 22.0 software (IBM SPSS, Chicago, IL) was used to analyze the data. Quantitative data were calculated as mean ± standard deviation (±SD). For data consistent with the normal distribution, the difference between two groups was compared using the independent samples t-test, and the difference between three or more groups was compared by analysis of variance (ANOVA). Pearson's correlation analysis was used to explore the relationship between the two variables. *P* < 0.05 was considered statistically significant.

## 3. Results

### 3.1 LOX expression was associated with invasion and metastasis of gastric cancer tissue

**Figure 1:**
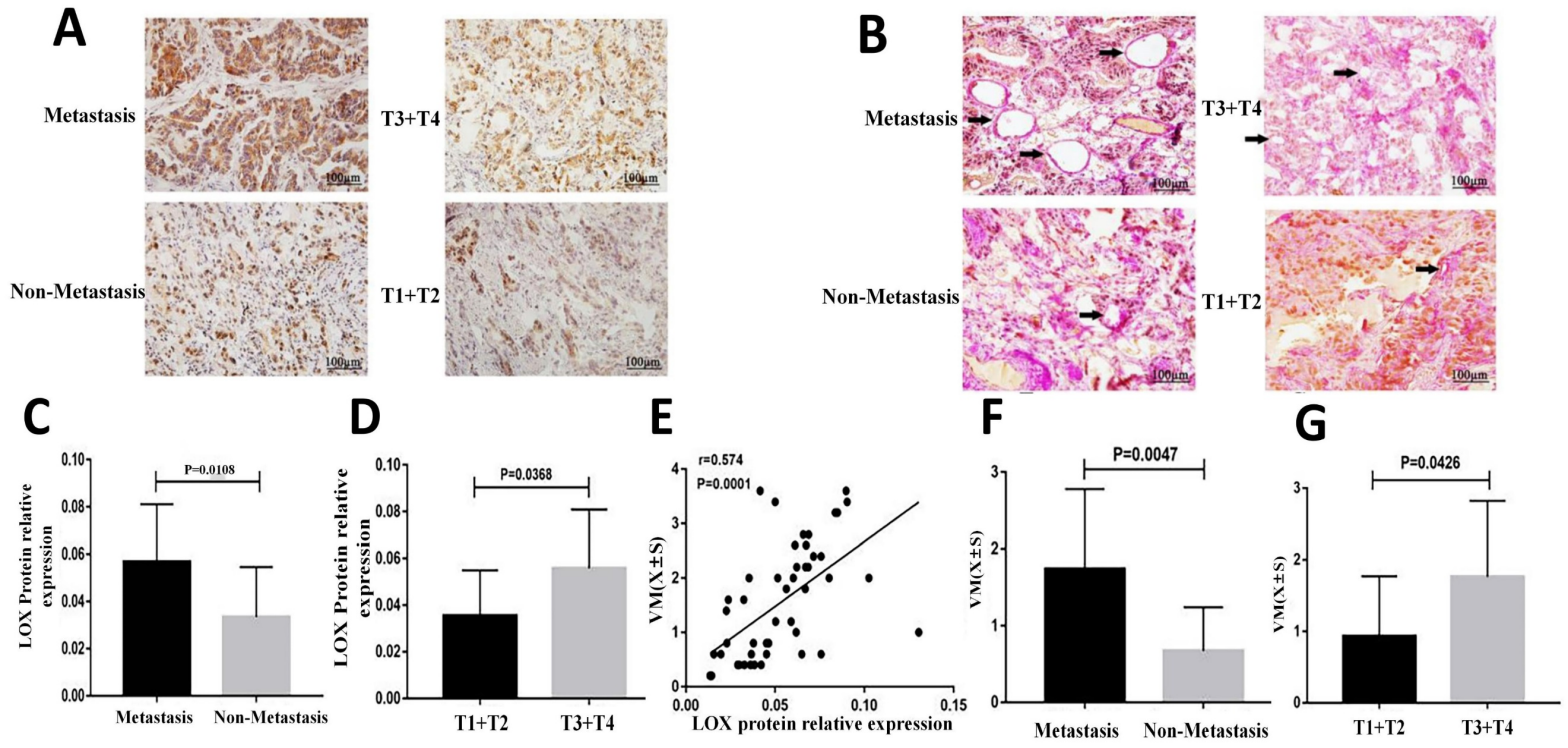
** Comparison of the level of LOX and the degree of VM in gastric cancer tissues. (A)** Images of LOX protein expression in gastric cancer tissues detected by immunohistochemistry(bars: 100 μm, brown indicates LOX positive);** (B)** Images showing anti-CD34 immunohistochemistry co-stained with a periodic acid-Schiff reagent to detect VM formation (bars: 100 μm); **(C)** Bar charts show that LOX MOD in metastatic gastric cancer tissues was significantly higher than in nonmetastatic gastric cancer tissues, P < 0.05; **(D)** Bar charts show that LOX MOD in gastric cancer tissues of the T3 + T4 group was significantly higher than of the T1 + T2, P < 0.05;** (E)** LOX relative expression was positively correlated with VM in gastric cancer tissues (r = 0.574, P <0.01). **(F)** Bar graphs show that the number of VM structures in metastatic gastric cancer was higher than in nonmetastatic gastric cancer, P < 0.05; **(G)** Bar graphs show that the number of VM structures in gastric cancer tissues in the T3+T4 group was higher than in the T1 + T2 group, P < 0.05. The error bars represent the standard deviation (SD).

LOX levels in 49 cases of gastric cancer were determined by immunohistochemistry. All gastric cancer tissues expressed LOX. The MOD of LOX in metastatic gastric cancer tissues was significantly higher than in nonmetastatic gastric cancer tissues (0.0568 ± 0.0244 vs. 0.0334 ± 0.0212), *P* < 0.05 (Table 1, Figure 1A and 1C). LOX MOD in gastric cancer samples in the T3-T4 group was significantly higher than in the T1-T2 group (0.0558 ± 0.0252 vs. 0.0355 ± 0.0195), *P* < 0.05 (Table 1, Figure 1A and 1D).

**Figure 2:**
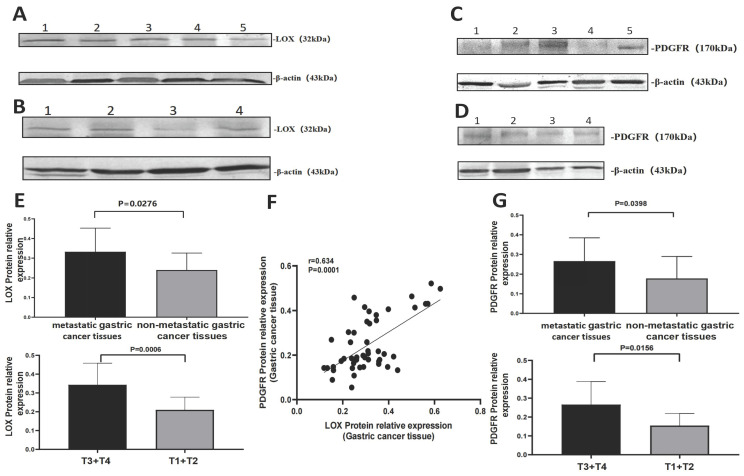
** Comparison of the relative expression of LOX and PDGFR in gastric cancer tissues through WB. (A)** Relative expression of LOX: metastatic gastric cancer tissues (Lanes 1-3), nonmetastatic gastric cancer tissues (Lanes 4-5). **(B)** Relative expression of LOX: T3-T4 group (Lanes 1-2), T1-T2 group (Lanes 3-4). **(C)** Relative expression of PDGFR: metastatic gastric cancer tissues (Lanes 1-3), nonmetastatic gastric cancer tissues (Lanes 4-5). **(D)** Relative expression of PDGFR: T3-T4 group (Lanes 1-2), T1-T2 group (Lanes 3-4). **(E)** Relative expression of LOX in metastatic gastric cancer tissues was higher than in nonmetastatic gastric cancer tissues; the relative expression of LOX in the T3-T4 group was higher than in the T1-T2 group, P < 0.05. **(F)** Scatter plot showing that the relative expression of LOX was positively correlated with PDGFR in gastric cancer tissue (**r**_LOX-PGGFR_ = 0.634, P < 0.0001). **(G)** Bar charts show that the relative expression of PDGFR in metastatic gastric cancer tissues was higher than in nonmetastatic gastric cancer tissues; the relative expression of PDGFR in the T3-T4 group was higher than in the T1-T2 group, P < 0.05.

Relative expression of LOX was also evaluated in 49 cases of gastric cancer by Western blotting. The relative expression of LOX in metastatic gastric cancer tissues was higher than in nonmetastatic gastric cancer tissues (0.33 ± 0.12 vs. 0.22 ± 0.07), *P* <0.05 (Table 2, Figure 2A and 2E). The relative expression of LOX in gastric cancer samples from the T3-T4 group was higher than in the T1-T2 group (0.33 ± 0.12 vs. 0.22 ± 0.07), *P* < 0.05 (Table 2, Figure 2B and 2E)**.**

### 3.2 VM formation was associated with invasion and metastasis of gastric cancer tissue and was positively correlated with LOX expression

To assess the relationship between VM formation and gastric cancer infiltration, we measured VM in 49 gastric cancer samples. The number of VM structures in gastric cancer tissues with metastasis was significantly higher than in gastric cancer tissues without metastasis (1.74 ± 1.04 vs. 0.67 ± 0.57), *P* < 0.05 (Table 1, Figure 1B and 1F). The number of VM structures in gastric cancer in the T3-T4 group was significantly higher than in the T1-T2 group (1.76 ± 1.06 vs. 0.93 ± 0.84), *P* < 0.05 (Table 1, Figure 1B and 1G).

Pearson's correlation analysis showed that the VM number was positively correlated with LOX expression (r = 0.574, *P* < 0.01; Figure 1E).

### 3.3 LOX promoted the formation of VM structures of gastric cancer cells

**Figure 3:**
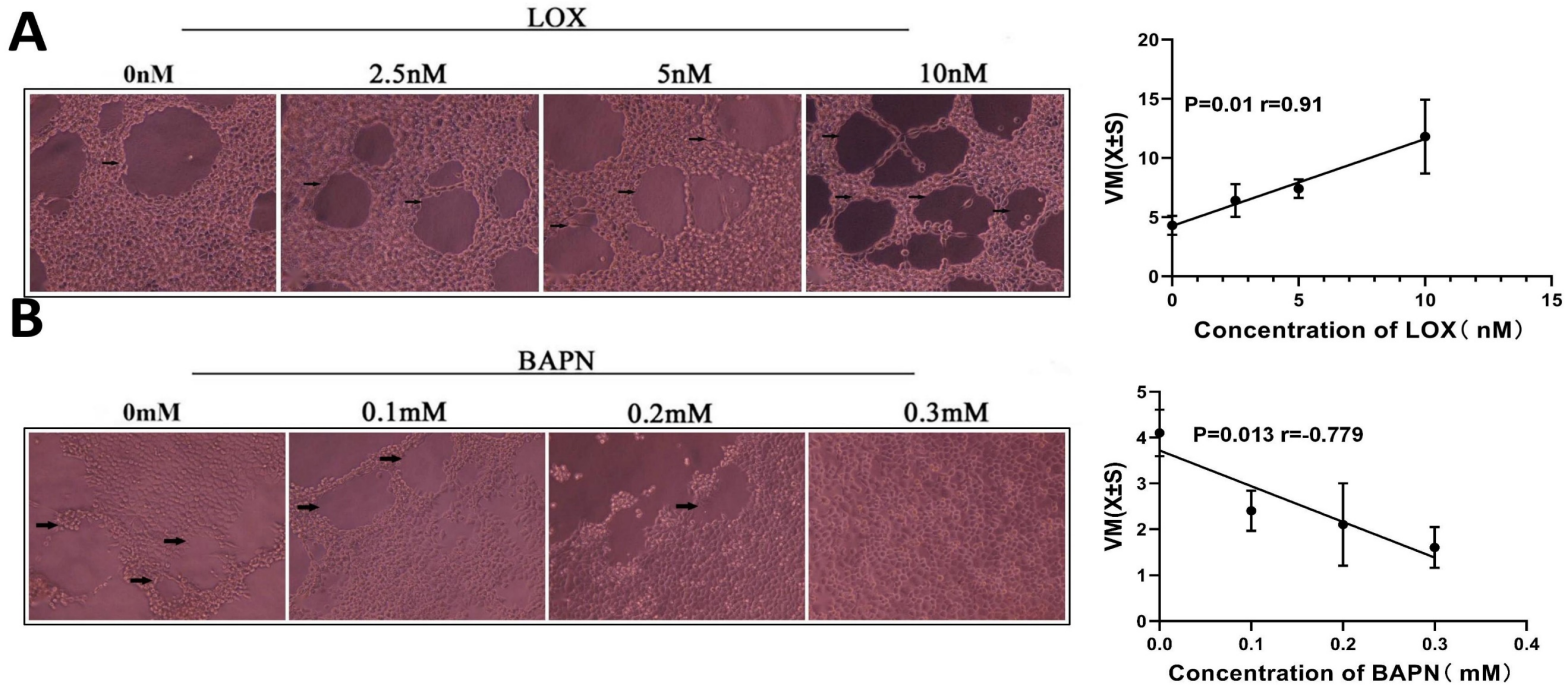
** LOX significantly improved VM formation in gastric cancer cells.** The VM were analyzed by three-dimensional culture of gastric cancer cells.** (A)** VM formation in gastric cancer cells treated with LOX. The LOX concentration was positively correlated with the number of VM structures. **(B)** VM formation in gastric cancer cells treated with BAPN. BAPN concentration was negatively correlated with the number of VM structures. The black arrow indicates VM formation (×40).

To evaluate LOX's effect on gastric cancer cell VM formation, we treated cells with different concentrations of LOX or BAPN. The number of VM structures increased with the LOX concentration (Figure 3A). LOX concentration was positively correlated with the number of VM structures (r_LOX/VM_ = 0.91, *P* < 0.05). On the contrary, the number of VM structures decreased with increasing BAPN concentration (Figure 3B). BAPN concentration was negatively correlated with the number of VM structures (r_BAPN/VM_ = -0.779, *P* < 0.05).

### 3.4 PDGFR was associated with invasion and metastasis of gastric cancer tissue and was positively correlated with LOX

We extracted proteins from gastric cancer tissues to determine the expression of PDGFR by Western blotting. The relative expression of PDGFR in metastatic gastric cancer tissues was greater than in nonmetastatic gastric cancer tissues (0.27 ± 0.12 vs. 0.15 ± 0.08), *P* < 0.01. The relative expression of PDGFR in gastric cancer samples in the T3-T4 group was higher than in the T1-T2 group (0.27 ± 0.12 vs. 0.15 ± 0.06), *P* < 0.01 (Table 2, Figure 2C, 2D, and 2G)**.**

The relative expression of PDGFR was positively correlated with LOX in gastric cancer tissue, r_LOX/PGGFR_=0.634, *P* < 0.0001 (Figure 2F).

### 3.5 LOX promoted the expression of PDGFR

**Figure 4:**
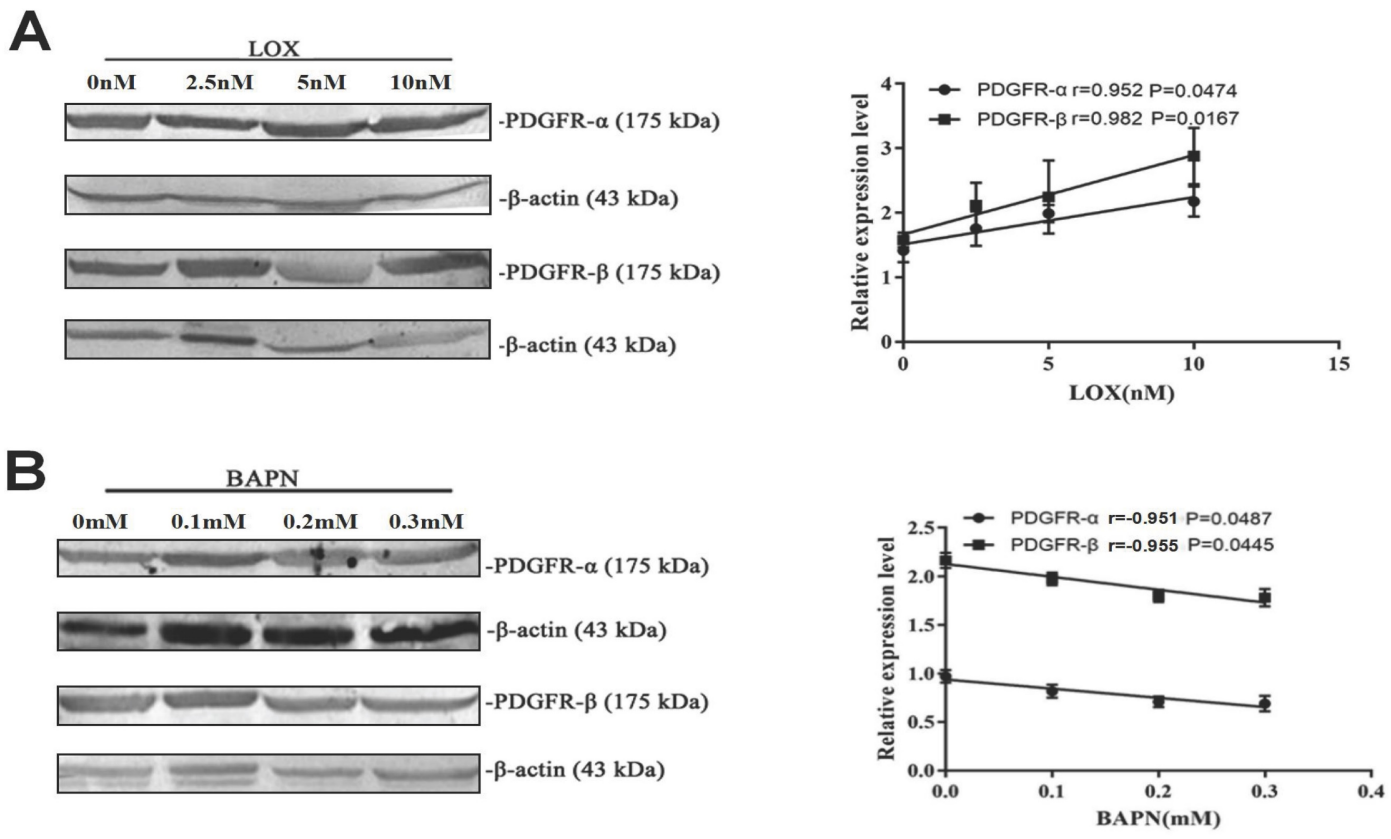
** LOX upregulated PDGFR expression in gastric cancer cells. (A)** Results of Western blot show the relative expression of PDGFRα and PDGFRβ in gastric cancer cells treated with exogenous LOX. The LOX concentration was positively correlated with the relative expression of PDGFR. **(B)** Western blot shows the relative expression of PDGFRα and PDGFRβ in gastric cancer cells treated with BAPN. The concentration of BAPN was negatively correlated with the relative expression of PDGFR. Error bars represent SD, n = 5.

To study the effect of LOX on the PDGF-PDGFR pathway, the relative expression of PDGFR-α and PDGFR-β in gastric cancer cells was measured after treatment with LOX or BAPN. The relative expression of PDGFR-α and PDGFR-β in gastric cancer cells was augmented with the increase in LOX concentration. LOX concentration was positively correlated with the relative expression of PDGFR (r_LOX/PDGFR/α_ = 0.952, r_LOX/PDGFR/β_ = 0.982, *P* < 0.05), and LOX promoted the expression of PDGFR α and β (Figure 4A). After BAPN treatment, the relative expression of PDGFR-α and PDGFR-β in gastric cancer cells was downregulated with increased BAPN concentration. BAPN concentration was negatively correlated with the relative expression of PDGFR (r_BAPN/PDGFR/α_ = -0.951, r_BAPN/PDGFR/β_ = -0.955, *P* < 0.05), and BAPN inhibits the expression of PDGFR α and β (Figure 4B).

### 3.6 PDGFR promoted the formation of VM structures of gastric cancer cells

**Figure 5:**
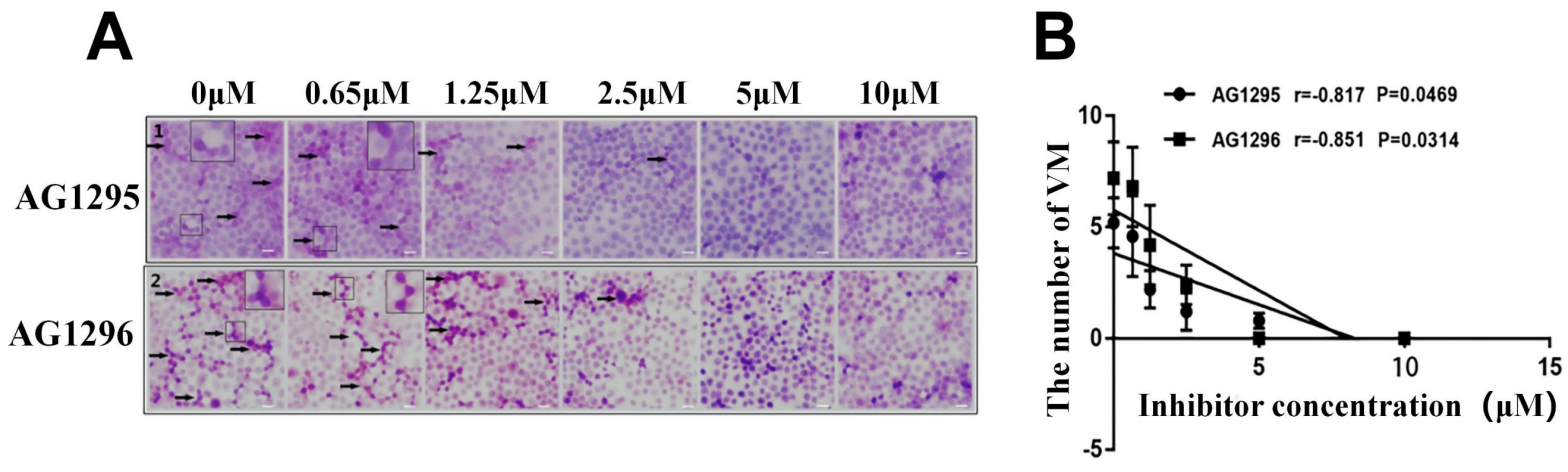
** PDGFR inhibitors can suppress VM formation in gastric cancer cells.** The VM were analyzed by two-dimensional culture of gastric cancer cells.** (A)** Different concentrations of PDGFR inhibitors significantly inhibited VM formation in gastric cancer cells (black arrow indicates VM formation; ×40); **(B)** Concentrations of PDGFR inhibitors were negatively correlated with the number of VM structures.

To evaluate the effect of PDGFR on VM formation by gastric cancer cells, gastric cancer cells were treated with PDGFR inhibitors AG1295 and AG1296. After inhibition with AG1295 or AG1296, the number of VM structures in gastric cancer cells decreased with increasing concentrations of AG1295 or AG1296 (Figure 5A). AG1295 and AG1296 concentrations were negatively correlated with the number of VM structures in gastric cancer cells (r_AG1295/VM_ = -0.817, r_AG1296/VM_ = -0.851, *P* <0.05, Figure 5B). The formation of VM in gastric cancer cells was completely inhibited after treatment with 10 μM AG1295 and 5 μM AG1296.

### 3.7 LOX enhanced VM formation, in part through the PDGF-PDGFR pathway

**Figure 6:**
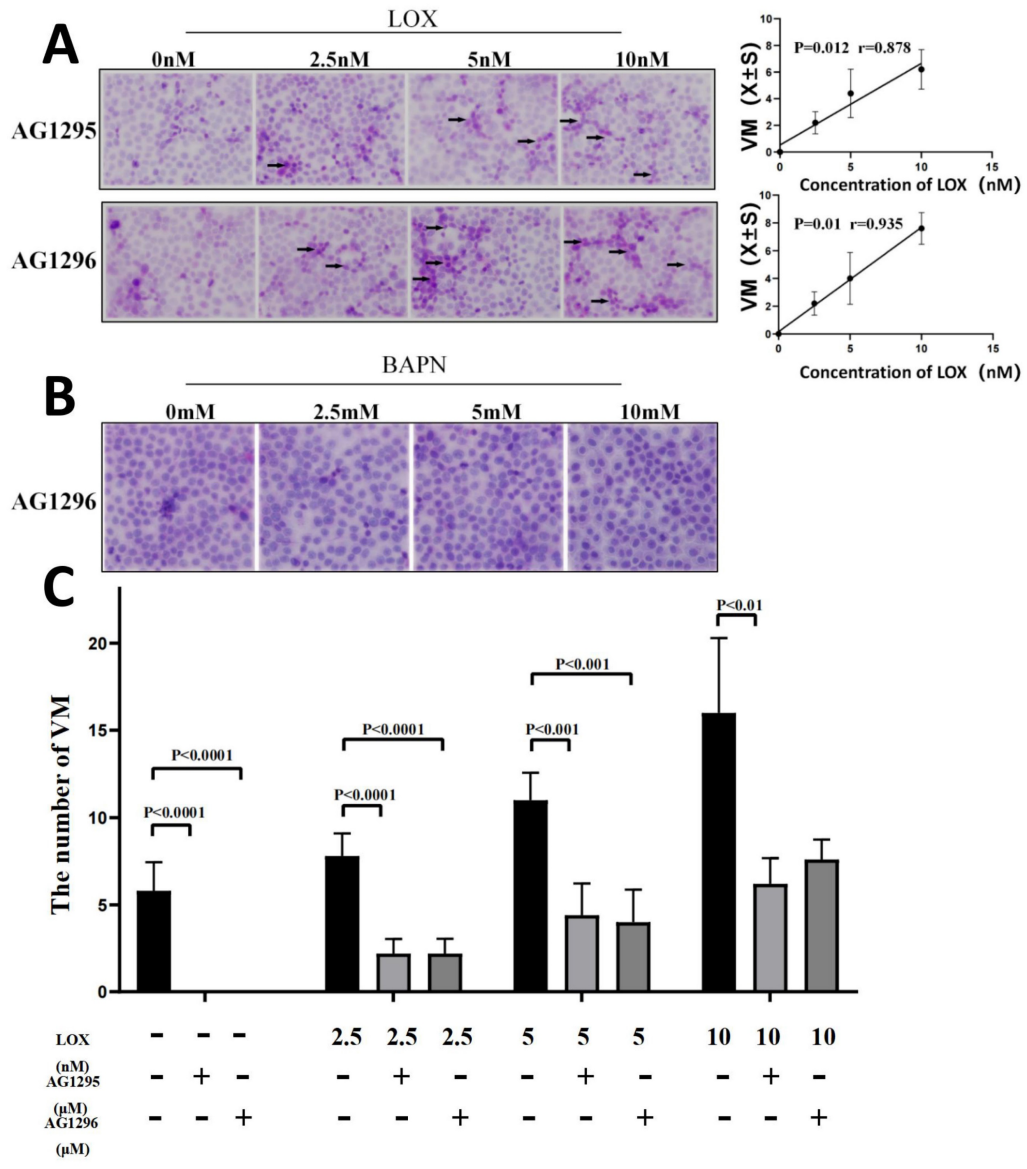
** Blocking PDGFR may partially inhibit the promotion of LOX on VM formation.** The VM were analyzed by two-dimensional culture of gastric cancer cells.** (A)** VM formation of gastric cancer cells treated with LOX and 10 μM AG1295 or 5 μM AG1296 (black arrow indicates VM formation; ×40). The LOX concentration was positively correlated with the number of VM structures.** (B)** VM formation of gastric cancer cells treated with BAPN and 5 μM AG1296. **(C)** Comparison of VM formation with or without AG1295 and AG1296 at the same concentration of LOX.

To assess whether LOX improved VM formation through the PDGF-PDGFR pathway, PDGFR was completely blocked in human gastric cancer cells using 10 μM AG1295 or 5 μM AG1296, while different concentrations of LOX were added. The number of VM structures in gastric cancer cells gradually increased with increasing LOX concentrations. LOX concentrations were positively correlated with the number of VM formations in human gastric cancer cells (r_AG1295/VM_ = 0.878, r_AG1296/VM_ = 0.935, *P* < 0.05, Figure 6A). When PDGFR was blocked by 5 μM AG1296 while different concentrations of BAPN were added, VM was not found in gastric cancer cells (Figure 6B).

The VM formation was then compared with or without AG1295 and AG1296 at the same concentration of LOX. The number of VM structures was significantly higher when no AG1295 or AG1296 was added to human gastric cancer cells (Figure 6C).

## 4. Discussion

Angiogenesis, the formation of new blood vessels, is a complex and dynamic process regulated by various pro- and anti-angiogenic molecules, which plays a crucial role in tumor growth, invasion, and metastasis. Since Folkman discovered in 1971 that the blood supply required for tumor growth depends on angiogenesis, angiogenesis has been considered the only way to supply blood for tumor growth. Inhibition of angiogenesis to further inhibit tumor growth and metastasis has become the main direction of antitumor therapy.

However, studies have shown that some therapeutic strategies targeting tumor blood vessels cannot completely inhibit tumor growth and metastasis, which may be related to the inability to inhibit VM formation 23-24. VM, discovered in 1999, is an important supplement to the blood supply pathway of tumors 6. VM is the process by which tumor cells form lumen-shaped structures in gastric cancer tissues, with or without red blood cells inside, exhibiting negative CD34 staining and positive PAS staining 12. VM is not only closely related to tumor biological behavior, such as tumor growth, invasion, and metastasis, but also negatively related to tumor prognosis. VM has become a new target for antitumor therapy 24-25. VM has been found to regulate various cytokines and enzymes, such as VEGF, MMP2, MMP9, EGF, and VE-cadherin 26. However, the effect of LOX on the formation of VMs and its mechanisms is still unclear.

This study explored LOX expression and the number of VM structures in clinical specimens of 49 patients with gastric cancer. Both LOX expression and VM formation promoted gastric cancer invasion and metastasis, and the LOX protein in gastric cancer tissues was positively correlated with the number of VM structures, indicating that the higher the expression of LOX, the more VM structures formed in gastric cancer tissues. To confirm this finding, we added exogenous LOX or LOX inhibitor (BAPN) to the gastric cancer cell line BGC-803 and observed VM formation during cell culture. The results found that the concentration of LOX was positively correlated with the VM number of gastric cancer cells. On the contrary, BAPN concentration was negatively correlated with the VM number of gastric cancer cells, indicating that LOX could promote VM formation in gastric cancer cells. The above results suggest that LOX may promote the formation of VM in gastric cancer, thereby accelerating the invasion and metastasis of gastric cancer.

The PDGF-PDGFR signaling pathway is closely related to biological behaviors, such as angiogenesis, invasion, and metastasis of various tumors 18-21. PDGFR is the receptor for PDGF with α and β subunits, which are divided into three subtypes of PDGFRαα, PDGFRαβ, and PDGFRββ through dimeric form, in which α has high affinity for the A, B, and C chains of the four polypeptide chains of PDGF. By contrast, β has a high affinity for the B and D chains. Studies have shown that the level of PDGFR expression is positively correlated with the degree of malignancy, and high expression of PDGFR is conducive to tumorigenesis and tumor progression 27,28. Tyrphostin AG1295, an inhibitor of the tyrosine kinase protein of PDGFR 29, significantly inhibits PDGFR-β mRNA (IC_50_ = 0.4 μM). The PDGFR inhibitor AG1296 competitively inhibits PDGFR through ATP 30, with an IC50 of 1 μM for PDGFRα and 0.8 μM for PDGFRβ.

To study the effect of the PDGF-PDGF R signaling pathway on VM formation, different concentrations of PDGFR inhibitors AG1295/AG1296 were used to treat gastric cancer cells. The results indicated a significant inhibition in VM formation in gastric cancer cells with the increase in AG1295 and AG1296. The concentrations of AG1295 and AG1296 were negatively correlated with the number of VM structures. Application of 10 μM AG1295 or 5 μM AG1296 completely inhibited the formation of VM structures in gastric cancer cells. This suggests that the PDGF-PDGFR signaling pathway was important in VM formation in gastric cancer cells. Blocking the PDGF-PDGFR signaling pathway can inhibit VM formation, and upregulating the expression of PDGFR in cancer cells can increase VM formation.

Lucero et al. have found that LOX activity is a prerequisite for PDGFR-mediated chemotaxis, and inhibition of LOX activity by BAPN can downregulate PDGF's chemotaxis and significantly inhibit signal transduction 22. To verify whether LOX regulates PDGFR expression in cells, different concentrations of exogenous LOX or BAPN were used to treat gastric cancer cells. The relative expression of PDGFRα and PDGFRβ in gastric cancer cells increased with increasing LOX concentration but decreased with increasing BAPN concentration. LOX concentration was positively correlated with the relative expression of PDGFR, and BAPN concentration was negatively correlated with the relative expression of PDGFR. Then, AG1295 (10 μM) or AG1296 (5 μM) concentrations were added to inhibit PDGFR function, and different concentrations of LOX or BAPN were added to detect VM formation in gastric cancer cells. The results revealed that LOX could still induce VM formation in gastric cancer cells when PDGFR was blocked, and LOX concentration was positively correlated with VM formation. However, when PDGFR was blocked, the VM formation induced by LOX was significantly less than that in PDGFR-unblocked cells. When PDGFR was completely blocked with AG1295 or AG1296, no VM was formed in MGC-803 cells, even when different concentrations of BAPN were added. These results indicate that LOX can upregulate the expression of PDGFR in gastric cancer cells and induce VM formation by upregulation of PDGFR. However, when the PDGFR function is blocked, LOX may promote VM formation through other mechanisms. These mechanisms need further study.

## 5. Conclusions

This study investigated the effect of LOX on VM formation in gastric cancer cells and its potential mechanism. We demonstrated that LOX can promote the formation of VM in gastric cancer and then promote the growth, invasion, and metastasis of gastric cancer. This mechanism is, in part, mediated through the PDGF-PDGFR signaling pathway. These mechanisms need to be further explored in future studies.

## Figures and Tables

**Table 1:** Comparison of LOX level and VM quantity in gastric cancer tissues

Group	Cases (N)	VM ( 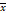 ± s)	MOD of LOX
T_1_+T_2_	8	0.93 ± 0.84	0.0355 ± 0.0195
T_3_+T_4_	41	1.76 ± 1.06^*^	0.0558 ± 0.0252^*^
Metastasis	40	1.74 ± 1.04	0.0568 ± 0.0244
Non-metastasis	9	0.67 ± 0.57^#^	0.0334 ± 0.0212^#^

^*^ P<0.05, T_3_+T_4_ vs T_1_+T_2_. ^#^ P < 0.05, non-metastasis vs. metastasis.

**Table 2:** Relative protein expression of LOX and PDGFR in gastric cancer tissues detected using Western blotting

Group	Cases (N)	LOX ( 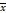 ± s)	PDGFR ( 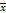 ± s)
T_1_+T_2_	8	0.22 ± 0.06	0.15 ± 0.06
T_3_+T_4_	41	0.33 ± 0.12^*^	0.27 ± 0.12^**^
Metastasis	40	0.33 ± 0.12	0.27 ± 0.12
Non-metastasis	9	0.22 ± 0.07^#^	0.15 ± 0.08^##^

^*^ P< 0.05; ^**^ P<0.01, T_3_+T_4_ vs T_1_+T_2_. ^#^ P < 0.05; ^# #^P<0.01, non-metastasis vs. metastasis.
